# A Translational Regulator, PUM2, Promotes Both Protein Stability and Kinase Activity of Aurora-A

**DOI:** 10.1371/journal.pone.0019718

**Published:** 2011-05-11

**Authors:** Yei-Hsuan Huang, Chun-Chi Wu, Chen-Kung Chou, Chi-Ying F. Huang

**Affiliations:** 1 Institute of Clinical Medicine, National Yang-Ming University, Taipei, Taiwan; 2 Institute of Medicine, Chung-Shan Medical University, Taichung, Taiwan; 3 Department of Life Science, Chang-Gung University, Tao-Yuan, Taiwan; Institut Pasteur, France

## Abstract

Aurora-A, a centrosomal serine-threonine kinase, orchestrates several key aspects of cell division. However, the regulatory pathways for the protein stability and kinase activity of Aurora-A are still not completely understood. In this study, PUM2, an RNA-binding protein, is identified as a novel substrate and interacting protein of Aurora-A. Overexpression of the PUM2 mutant which fails to interact with Aurora-A, and depletion of PUM2 result in a decrease in the amount of Aurora-A. PUM2 physically binds to the D-box of Aurora-A, which is recognized by APC/C^Cdh1^. Overexpression of PUM2 prevents ubiquitination and enhances the protein stability of Aurora-A, suggesting that PUM2 protects Aurora-A from APC/C^Cdh1^-mediated degradation. Moreover, association of PUM2 with Aurora-A not only makes Aurora-A more stable but also enhances the kinase activity of Aurora-A. Our study suggests that PUM2 plays two different but important roles during cell cycle progression. In interphase, PUM2 localizes in cytoplasm and plays as translational repressor through its RNA binding domain. However, in mitosis, PUM2 physically associates with Aurora-A to ensure enough active Aurora-A at centrosomes for mitotic entry. This is the first time to reveal the moonlight role of PUM2 in mitosis.

## Introduction

Aurora-A is a centrosomal serine-threonine kinase that regulates many processes during cell division. Both the expression and kinase activity of Aurora-A are tightly cell cycle-regulated, peaking when cells enter the M phase [1]. Mechanistically, the best understood roles of Aurora-A relate to the recruitment and regulation of proteins at the centrosomes. Aurora-A and its substrates complete the assignments in cell division coordinately through phosphorylation reactions, such as centrosome maturation, spindle assembly, mitotic commitment and cytokinesis [2]. Instead of serving as downstream effectors, some Aurora-A substrates and interacting proteins such as the microtubule-associated protein TPX2 [3], [4], the LIM domain-containing protein Ajuba [5] and the focal-adhesion protein PAK1 [6], play roles as upstream regulators that mediate Aurora-A kinase activity. Aurora-A activity is regulated by auto- or transphosphorylation at a conserved threonine residue (threonine-288 in humans) [1], [7]. Ajuba is involved in Aurora-A autophosphorylation, whereas PAK1 phosphorylates Aurora-A at threonine-288. Otherwise, upon binding to TPX2, a global conformational change of Aurora-A is triggered and the domain that contains phosphorylated threonine-288 enters a phosphatase-inaccessible conformation, increasing the kinase activity of Aurora-A [4]. Upon exit from mitosis, the Aurora-A kinase activity rapidly declines, and the protein is recognized by E3 ubiquitin ligase, APC/C^Cdh1^, and degraded by the ubiquitin-proteosome dependent mechanism [8], [9], [10], [11]. However, the regulatory pathways for kinase activity and the protein concentration of Aurora-A in cells are still not completely understood. Most of the reported Aurora-A substrates and interacting protein partners (which are Aurora-A regulators) act as kinase-activating factors when the cells enter the M phase, yet none have been identified as a factor that enhances the protein stability of Aurora-A. Besides, the expression of Aurora-A at the transcriptional level only increases to a small extent at the G2/M transition [12]. It remains to be elucidated how the protein quantity of Aurora-A can peak so dramatically, causing mitotic entry.

PUM2 (Human Pumilio homology protein 2), which is a member of the PUF protein family, has been reported to bind to 3′ untranslated regions (3′ UTRs) of mRNA and to repress gene expression by controlling cytoplasmic polyadenylation and affecting mRNA translation [13], [14], [15]. Interestingly, several studies on the maturation of *Xenopus* oocytes reveal that Aurora-A is also involved in this regulation and that PUM2 contributes to a selective translational repression of maternal cyclin B1 mRNA [16], [17]. When translationally dormant, PUM2, CPEB (CPE-binding protein) and Maskin form a complex on maternal cyclin B1 mRNA, precluding the formation of the active translation initiation complex. Aurora-A then promotes poly(A) elongation by phosphorylating CPEB, causing dissociation of the PUM2-CPEB-Maskin-eIF4E complex [18], [19], [20], [21]. Surprisingly, Maskin has been identified as the homologue of TACC3, a well-characterized substrate of Aurora-A [22]. TACC3 is required for microtubule assembly during the M-phase and is localized at the centrosomes, as regulated by Aurora-A [23]. Moreover, CPEB is present on the mitotic apparatus [24]. Two components of the protein complex that are involved in regulating the translation of *Xenopus* maternal cyclin B1 mRNA are substrates of Aurora-A and both of these also have roles in cell division. This raises the possibility that PUM2 might also have a second role in controlling the progression of cell cycle like Maskin and CPEB.

Here we demonstrate that PUM2 is a novel substrate of and binds to Aurora-A. Binding to PUM2 protects Aurora-A from the protein ubiquitination/degradation mediated by APC/C^Cdh1^, and that is required for the dramatic increase in the kinase activity of Aurora-A, causing mitotic entry. This study reveals that PUM2 plays an additional role in mitotic control, apart from its role in translational regulation during interphase. This finding supports the notion that cells employ the same components used in interphase to regulate mitosis.

## Results

### PUM2 is a substrate of Aurora-A kinase and its expression is cell cycle-regulated

**Figure 1:**
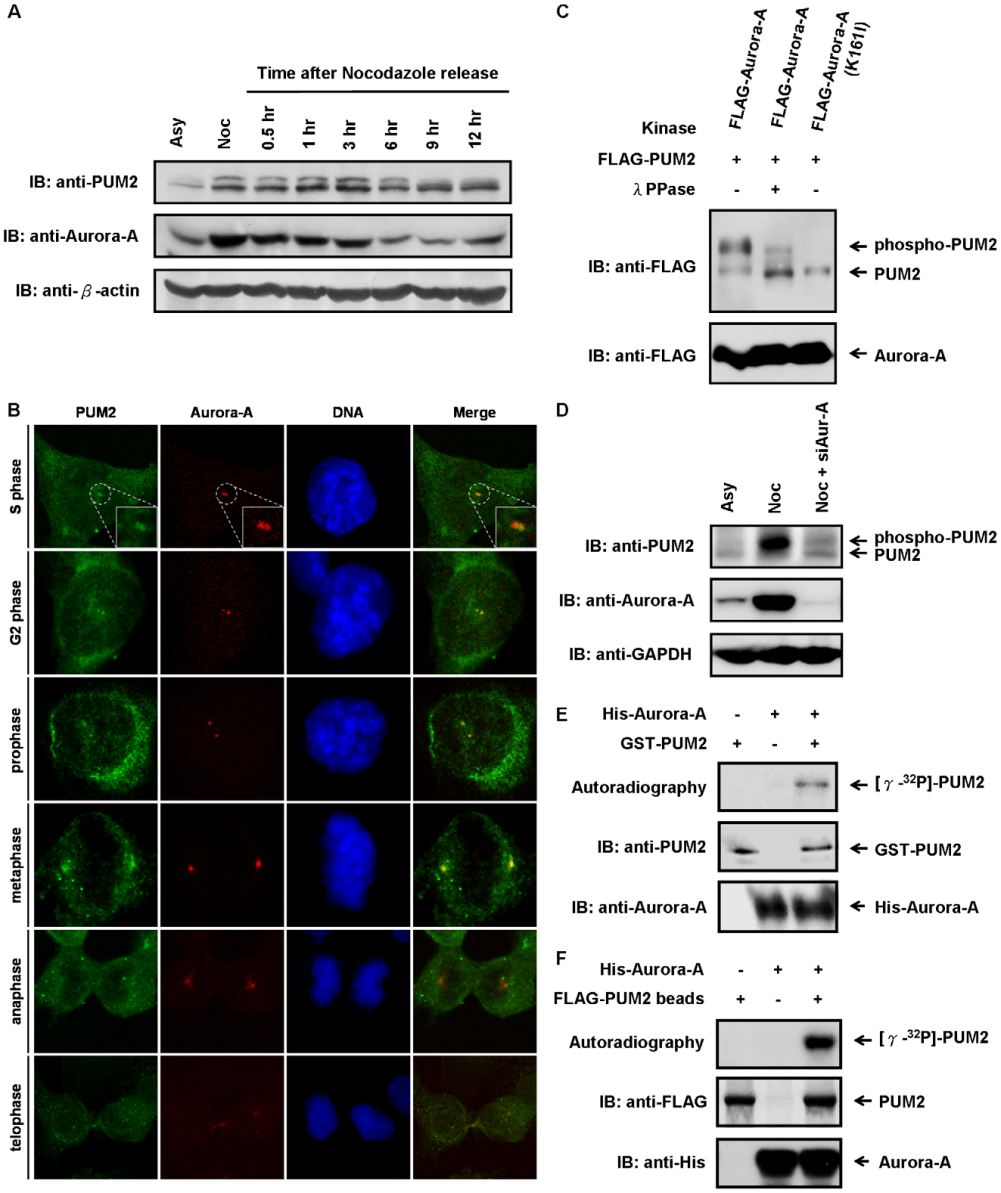
The cell cycle-regulated protein, PUM2, is a novel substrate of Aurora-A kinase. (A) PUM2 exhibites remarkably variations both in protein quantity and phosphorylation state during exit from the G2/M block. HeLa cells were synchronized in the G2/M phase by treatment with nocodazole for 16 hrs and subsequently released into cell cycle progression by removal of the nocodazole. At the indicated time points, the cells were harvested and analyzed by immunoblotting. Asynchronously (Asy) growing cells were analyzed in parallel. (B) PUM2 was localized at the centrosomes from S phase to metaphase. The CL_1–5_ cells were fixed and probed with anti-PUM2 antibody (green) and anti-Aurora-A antibody (red), and the DNA was stained with DAPI (blue). The cells were visualized using confocal fluorescence microscopy. (C) PUM2 is a novel substrate for Aurora-A. HEK293T cells were transfected with FLAG-tagged PUM2 in combination with FLAG-tagged Aurora-A (the wild-type or kinase-inactive mutant). To confirm whether the gel mobility up-shift was derived from phosphorylated PUM2, the cell lysates were treated with and without λ protein phosphatase. (D) The M phase-specific electrophoretic mobility shift of PUM2 is abolished in Aurora-A-depleted cells. HeLa cells were transfected with Aurora-A specific siRNA (siAur-A) and synchronized in the G2/M phase by treatment with nocodazole for 16 hrs. The cells were harvested and analyzed by immunoblotting. (E, F) PUM2 is an *in vitro* substrate of Aurora-A. GST-tagged PUM2 (E) or FLAG-tagged PUM2 immunoprecipitated from cell lysates (F) was incubated, either alone or in combination with recombinant His-tagged Aurora-A, in the presence of [γ-32P]-ATP. The samples were electrophoresed using SDS-PAGE and transferred to a PVDF membrane. They were then either autoradiographed or immunoblotted.

To test the hypothesis that PUM2 may have roles in the regulation of the progression of cell cycle, and that it is functionally linked to the mitotic kinase Aurora-A, we first determined whether the protein expression and intracellular localization of PUM2 was cell cycle-regulated. As compared to asynchronous cells, high levels of Aurora-A were detected in nocodazole-arrested mitotic cells, in line with previous observations [1] (Figure 1A). In addition, high levels of PUM2 were observed in these cells. Interestingly, PUM2 also exhibited an electrophoretic mobility up-shift, possibly caused by phosphorylation (Figure 1A). After release from nocodazole arrest, the protein level of PUM2 went down and its electrophoretic mobility up-shift disappeared in parallel to the concentration of Aurora-A in cells (Figure 1A). Moreover, PUM2 and Aurora-A colocalized to the centrosomes from S phase to metaphase (Figure 1B). These data suggest that PUM2 is functionally linked to Aurora-A.

To determine whether Aurora-A can phosphorylate PUM2 and hence lead to its electrophoretic mobility up-shift, FLAG-tagged PUM2 and wild-type Aurora-A or kinase-dead Aurora-A-K162I were cotransfected into HEK293T cells for analysis. In the presence of wild-type Aurora-A, PUM2 exhibited an apparent electrophoretic mobility up-shift, and the up-shift could be abolished by λ protein phosphatase. In contrast, in the presence of Aurora-A-K162I, PUM2 did not show the electrophoretic mobility up-shift (Figure 1C). The electrophoretic mobility up-shift of PUM2 was also examined by performing immunoprecipitation using anti-FLAG antibody to precipitate FLAG-tagged PUM2 from cell lysates followed by λ protein phosphatase treatment with or without protein phosphatase inhibitor, and similar phenomenon was observed (Figure S1). Moreover, in Aurora-A-depleted cells, the M phase-specific electrophoretic mobility shift of PUM2 was abolished (Figure 1D). These results suggest that PUM2 is a mitotic phosphoprotein and its phosphorylation is dependent upon Aurora-A. To confirm this, we performed *in vitro* kinase assay. Recombinant GST-tagged PUM2 or FLAG-tagged PUM2 immunoprecipitated from cell lysates was incubated either alone or in combination with His-tagged Aurora-A in a kinase buffer containing [γ-^32^P]-ATP. Phospho-PUM2 radioactivity was detected only in the presence of Aurora-A (Figure 1E, Figure 1F and Figure S2). Taken together, we conclude that PUM2 is a novel substrate of Aurora-A.

### The novel role of PUM2 in cells apart from the well-known role in the regulation of translation: to stabilize Aurora-A through physical interaction

**Figure 2:**
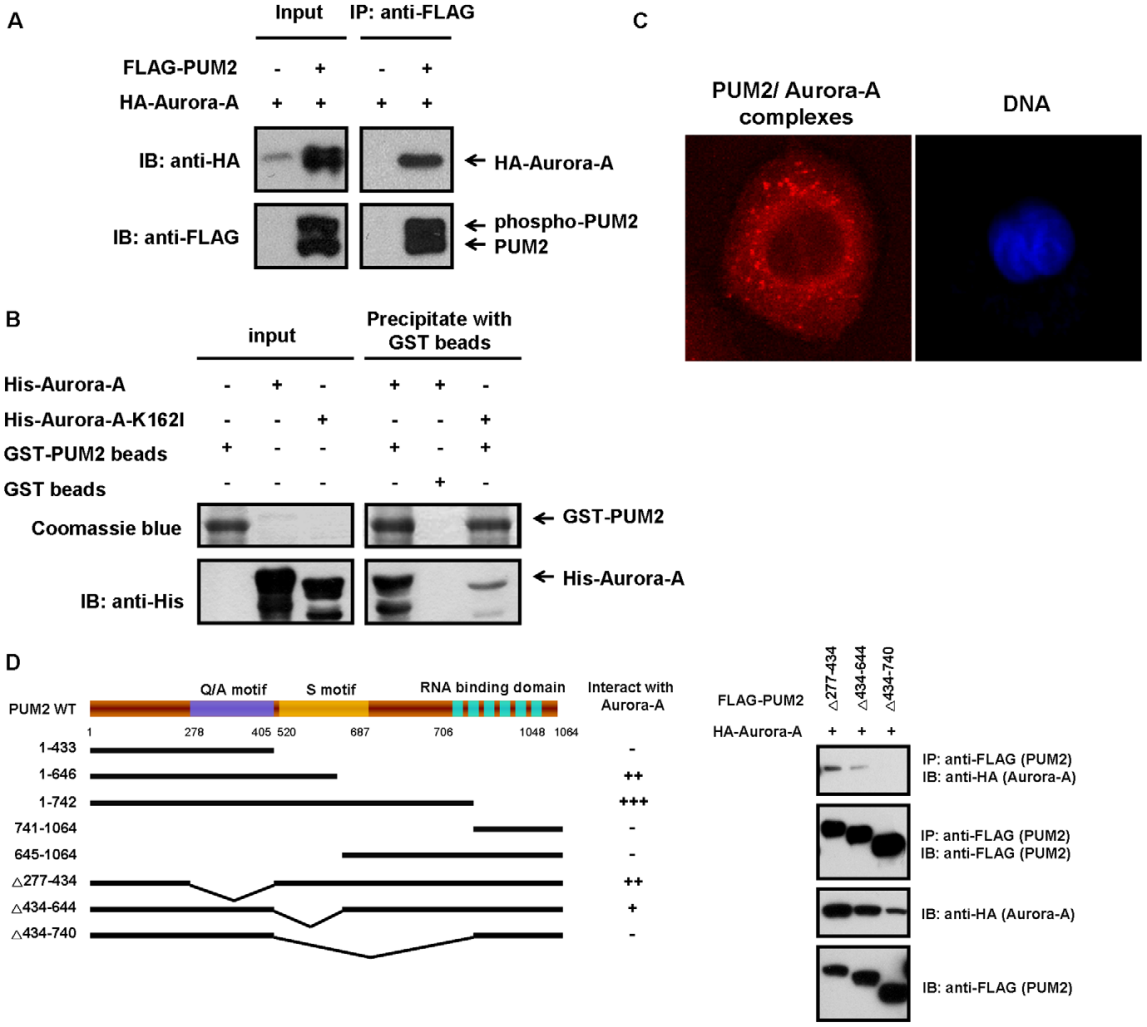
PUM2 interacts physically with Aurora-A, and the S motifs, not the PUM-HD motif, of PUM2 are required for this interaction. (A) PUM2 forms a complex with Aurora-A in HEK293T cells. HEK293T cells were transfected with HA-tagged Aurora-A, either alone or in combination with FLAG-tagged PUM2. The samples were immunoprecipitated using an anti-FLAG antibody and then immunoblotted with an anti-HA antibody to detect Aurora-A. (B) Recombinant Aurora-A and PUM2 form a complex as determined using a GST pull down assay. Purified GST-PUM2 fusion protein or a control (GST immobilized on glutathione-Sepharose 4B beads) was incubated with purified His-tagged Aurora-A or kinase-inactive mutant protein (Aurora-A-K162I). Bead-bound proteins were immunoblotted against an anti-His antibody, and the gel was also stained with coomassie blue. His-tagged Aurora-A and kinase-inactive mutant protein alone are also shown. (C) Detection of endogenous PUM2-Aurora-A complexes by *in situ* proximity ligation assay (PLA). Complexes between endogenous PUM2 and Aurora-A were visualized by staining CL_1–5_ cells with anti-PUM2 and anti-Aurora-A antibodies. Each red dot represented an interaction detected by the PLA assay. DNA was stained with DAPI (blue) and the cells were visualized using confocal fluorescence microscopy. (D) The S motifs of PUM2 are required for the interaction with Aurora-A. Five PUM2 truncation mutants and three deleted PUM2 mutants were generated based on the putative domains present in PUM2. HEK293T cells were transfected with HA-tagged Aurora-A and various FLAG-tagged PUM2 mutants. The cell lysates were immunoprecipitated with an anti-FLAG antibody and then immunoblotted with an anti-HA antibody to detect Aurora-A.

Given the colocalization of PUM2 with Aurora-A, we determined whether PUM2 and Aurora-A were present in the same complex. Therefore, HEK293T cells were transfected with HA-tagged Aurora-A, either alone or in combination with FLAG-tagged PUM2. By performing co-immunoprecipitation using anti-FLAG antibody and immunoblot analysis using the anti-HA antibody, Aurora-A was co-precipitated with PUM2 from cell lysates, suggesting that PUM2 interacts with Aurora-A either directly or via intermediate proteins (Figure 2A). To determine whether PUM2 directly interacted with Aurora-A, we performed an *in vitro* GST pull down assay. Bacterially-expressed, purified GST-PUM2 fusion protein or GST protein on glutathione-Sepharose 4B beads was incubated with purified His-tagged wild-type Aurora-A or kinase-inactive mutant protein (Aurora-A-K162I). We found that GST-PUM2 could bind to His-tagged Aurora-A, whereas GST could not (Figure 2B), indicating that PUM2 directly binds to Aurora A. In addition, it is worthy to note that the kinase activity of Aurora-A defective mutant (Aurora-A-K162I) showed low binding affinity with PUM2 comparing with the wild-type Aurora-A. This suggests that the binding of these two proteins is positively related to the kinase activity of Aurora-A. PUM2 might first be phosphorylated by Aurora-A and this phosphorylation promotes the binding between these two proteins. It was in further detail to investigate if endogenous PUM2 could interact with Aurora-A by the application of the *in situ* proximity ligation assay (PLA), that enables the detection and quantification of protein–protein interactions in native cells [25]. Complexes between endogenous PUM2 and Aurora-A were visualized by staining CL_1–5_ cells with anti-PUM2 and anti-Aurora-A antibodies, where individual primary antibody was performed as negative controls. The distance between the two primary antibodies needs to be less than 40 nm for the PLA assay to generate a red dot signal, which represents an interaction. Results shown in Figure 2C clearly indicates the presence of PUM2/Aurora-A complexs signals, revealing endogenous PUM2 could interact with Aurora-A.

Three putative domains including the S motif (serine rich regions), the Q/A motif (glutamine/alanine-rich regions) and the PUM-HD motif (RNA-binding domain), are present in PUM2 [13]. Five PUM2 truncation mutants were thus generated based on these motifs (left panel of Figure 2D). By performing co-immunoprecipitation assay, we examined the abilities of these PUM2 truncation mutant proteins to associate with Aurora-A. The results showed that the N-terminal fragment (aa 1–742 and aa 1–646) that contained the complete Q/A and S motifs had the strongest affinity for Aurora-A. The N-terminal fragment (aa 1–433), which included the Q/A motif but not the S motif, had a very low affinity for Aurora-A. However, various fragments containing different truncated C-terminal derivatives (aa 741–1064 and aa 645–1064) also had weak affinities for Aurora-A (Figure 2D). This revealed the S motif of PUM2 is required for the interaction with Aurora-A. To further verify the Aurora-A-binding region in PUM2, we generated three deleted PUM2 mutants, PUM2Δ277–434, lacking the Q/A motif, PUM2Δ434–644, lacking the S motif and PUM2Δ434–740, lacking the S motif and the sequence preceding the PUM-HD motif. PUM2Δ277–434 and PUM2Δ434–644 were able to bind to Aurora-A, but PUM2Δ434–740 was not (Figure 2D and Figure S3), supporting the idea that the S motif of PUM2 is responsible for binding to Aurora-A. Moreover, the sequence preceding the PUM-HD motif of PUM2 is also important for this interaction. In previous study, the PUM-HD motif of PUM2 is responsible for the well-known role as translational repressor [14]. However, our results showed that the N-terminal fragment (aa 1–742) which lacked the PUM-HD motif still could bind to Aurora-A and another motif, the S motif, is responsible for this interaction. This revealed that PUM2 might have the novel role in cells apart from the well-known role in the regulation of translation.

**Figure 3:**
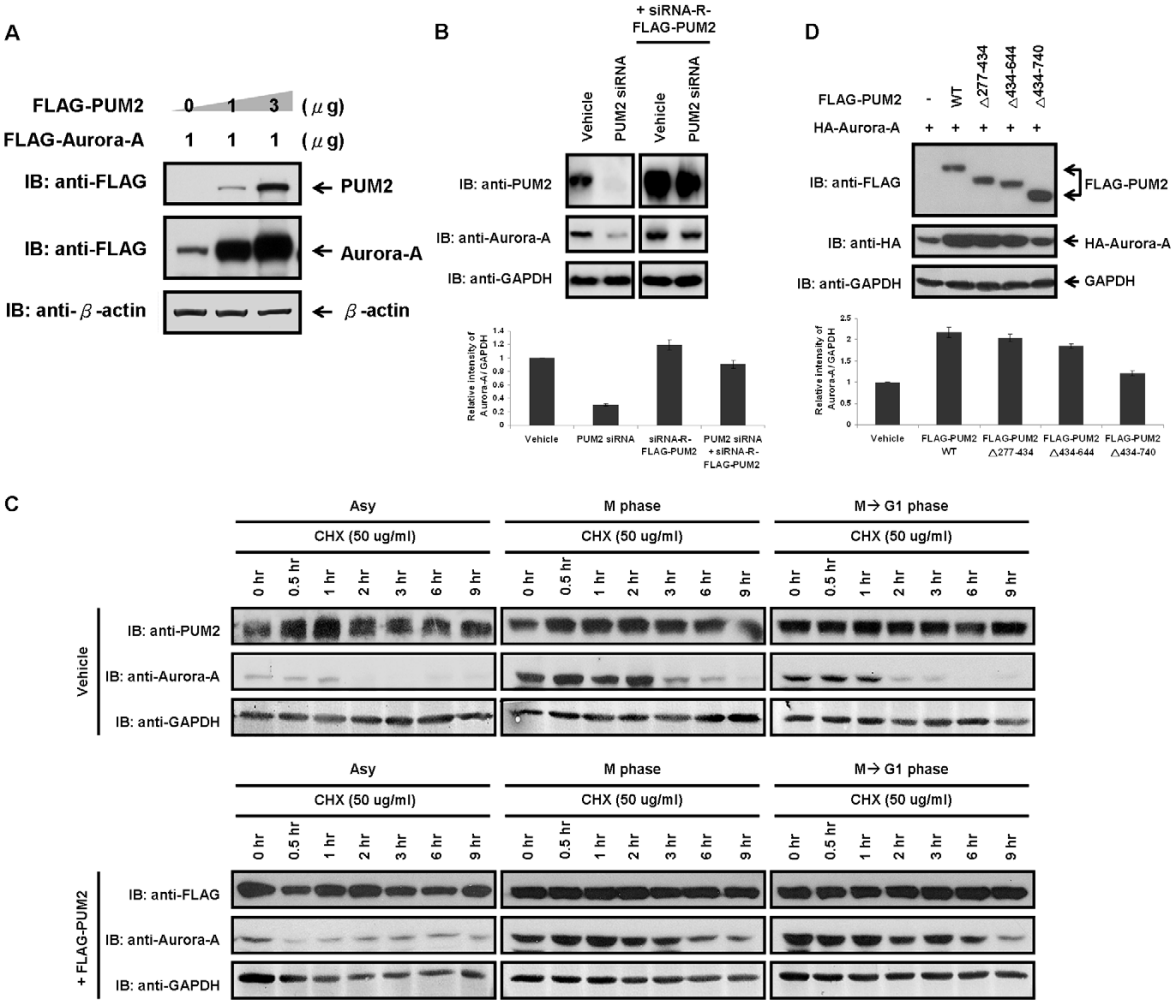
PUM2 is able to enhance the protein stability of Aurora-A, which is indispensable for the interaction between Aurora-A and PUM2. (A) The stability of Aurora-A is regulated by PUM2. HEK293T cells were transfected with FLAG-tagged Aurora-A, either alone or with different amounts of FLAG-tagged PUM2. The cell lysates were analyzed by immunoblotting. (B) Knock-down of PUM2 results in the down-regulation of both PUM2 and Aurora-A. HEK293T cells were transfected with PUM2 specific siRNA or in combination with siRNA-R-FLAG-PUM2 and the effect of steady-state protein level of Aurora-A was determined using immunoblotting analysis. The relative intensity of protein bands on immunoblotting were quantified and normalized to GAPDH. (C) PUM2 protects Aurora-A from protein degradation when the cells exit from mitosis. HEK293T cells were transiently transfected with an empty vector or with FLAG-tagged PUM2. 24 hrs after transfection, the cells were synchronized in mitosis by nocodazole. For the collection of cells exiting from M phase, the cells were released into the cell cycle progression by removing the mitosis-synchronizing reagent and subsequently incubated with fresh medium containing cycloheximide, which blocks *de novo* protein synthesis. For the collection of M phase cells, the cells were also treated with nocodazole but this mitosis-synchronizing reagent was not removed when incubating with cycloheximide-containing medium. At the indicated time points, the cells were harvested and the lysates were immunoblotted with anti-PUM2, anti-FLAG, anti-Aurora-A and anti-GAPDH antibodies. Asynchronously (Asy) growing cells were analyzed in parallel. (D) Overexpression of PUM2 mutant, which failed to interact with Aurora-A, leads to the destabilization of this kinase.

Of particular interest is that the amount of ectopically expressed Aurora-A proteins was higher in cells transfected with Aurora-A alone than in cells co-expressed with PUM2 and Aurora-A (left panel of Figure 2A). It raises the possibility that PUM2 might stabilize Aurora-A protein and hence result in an accumulation of Aurora-A. To test this possibility, we investigated whether increasing amounts of PUM2 correlate with increased steady-state protein level of Aurora-A. We co-expressed Aurora-A (1 µg) with different amounts of PUM2, and immunoblotting analysis revealed that the increase in the protein amount of Aurora-A was accompanied by a substantial dose dependent increase in the steady-state protein amount of PUM2 (Figure 3A). Consistent with this hypothesis, the depletion of endogenous PUM2 by siRNA also resulted in a decrease in the steady state level of Aurora-A (left panel of Figure 3B). To further examine the specificity of PUM2 siRNA, the rescue assay with siRNA-resistant FLAG-PUM2 (siRNA-R-FLAG-PUM2) was performed. Three nucleotides of wild-type PUM2 were mutated to create resistance to PUM2 siRNA, and the encoding protein was not altered. It was shown the effect of PUM2 siRNA in the steady state level of Aurora-A is restored by re-expressing the RNAi-resistant FLAG-PUM2 (right panel of Figure 3B).

To determine whether PUM2 stabilize Aurora-A at protein level, we examined the effect of PUM2 on the protein turnover rate of endogenous Aurora-A. The cycloheximide (CHX) chase experiment was performed using cell lysates from nocodazole-arrested M-phase cells and late anaphase/early G1-phase cells that were released from nocodazole block. It was shown that Aurora-A was stabilized in M-phase cells overexpressing PUM2 and had a half-life of longer than 3 hours but it had a half-life of less than 3 hours in cells transfected with empty vector (middle panel of Figure 3C). Similarly, after release from nocodazole arrest, Aurora-A was highly stabilized in cells overexpressing PUM2, with a half-life of longer than 6 hours as compared to a half-life of less than 1 hour in cells transfected with the empty vector (right panel of Figure 3C). These results indicate that Aurora-A is rapidly degraded during mitotic exit and this degradation can be prevented by overexpression of PUM2.

To explore how PUM2 stabilizes Aurora-A, we determined whether the interactions between Aurora-A and PUM2 are necessary for modulating the protein stability of Aurora-A. If these two events are correlated, Aurora-A binding-deficient mutant(s) of PUM2 should lose the ability to stabilize Aurora-A. As compared to cells transfected with wild-type PUM2 or the PUM2 mutant proteins which could not interact with Aurora-A, PUM2Δ434–740, the protein level of Aurora-A was much lower in cells transfected with this PUM2 deleted mutant protein (Figure 3D). These indicated that binding of PUM2 to Aurora-A is required to stabilize Aurora-A. Taken together, these results led to the conclusion that PUM2 is required to maintain sufficient amounts of Aurora-A in the cells, which is indispensable for the interaction between Aurora-A and PUM2.

### Binding of PUM2 to Aurora-A protects Aurora-A from APC/C^Cdh1^-mediated degradation

It is known that the ubiquitin ligase APC/C^Cdh1^ targets the amino-terminal A-box and carboxy-terminal D-box sequences of Aurora-A for proteolysis. Consistent with previous observation [10], when Aurora-A was co-expressed with different amounts of Cdh1 in HEK293T cells, the increase in the protein level of Cdh1 was accompanied by a substantial decrease in the expression levels of Aurora-A. However, overexpression of Cdc20 did not affect the expression level of Aurora-A (Figure S4).

**Figure 4:**
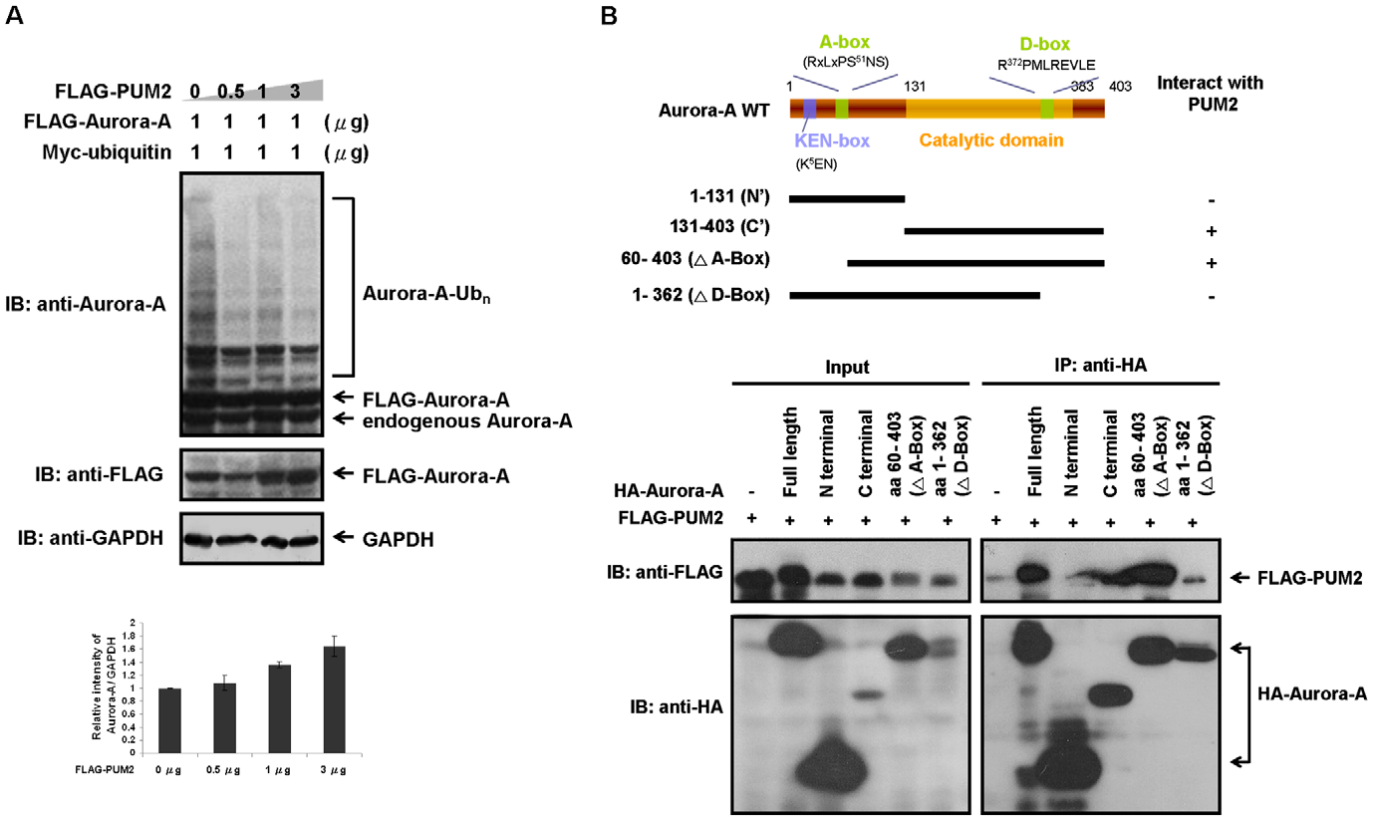
Binding of PUM2 to Aurora-A protects Aurora-A from APC/C^Cdh1^-mediated degradation. (A) PUM2 blocks the ubiquitination of Aurora-A. HEK293T cells were transfected with FLAG-tagged Aurora-A, together with different amounts of FLAG-tagged PUM2. Myc-tagged ubiquitin was also added to reveal the ubiquitination of Aurora-A. 24 hrs after transfection, the cells were synchronized in the G2/M phase by treatment with nocodazole for 16 hrs. Subsequently, the synchronized cells were released into cell cycle progression in the presence of a proteasome inhibitor (MG132) for 9 hrs. High molecular weight ubiquitinated Aurora-A accumulated in the transfected cells that were treated with MG132, as shown. The relative intensity of protein bands represented the steady-state protein level of Aurora-A on immunoblotting analysis were quantified and normalized to GAPDH. (B) The D-box of Aurora-A mediates its association with PUM2. HEK293T cells were transfected with FLAG-tagged PUM2 and various HA-tagged Aurora-A fragments. The cell lysates were immunoprecipitated with an anti-HA antibody and immunoblotted with an anti-FLAG antibody to detect PUM2.

Since the association between Aurora-A and PUM2 is necessary for enhancing the stability of Aurora-A, PUM2 should first physically interact with Aurora-A and then modulate its protein stability. We reasoned that PUM2 should prevent Aurora-A from APC/C^Cdh1^-dependent ubiquitination directly. It was expected that Aurora-A might be less susceptible to ubiquitination in cells when PUM2 is overexpressed. Different amounts of FLAG-tagged PUM2 were co-expressed with FLAG-tagged Aurora-A and Myc-tagged ubiquitin in HEK293T cells. 24 hrs after transfection, cells were treated with the proteasome inhibitor MG132 to allow accumulation of ubiquitinated forms of Aurora-A at the timepoint when G2/M-arrested cells were released into the cell cycle progression. As expected, Aurora-A was less susceptible to ubiquitination in cells in which PUM2 was overexpressed (Figure 4A). Increasing the protein level and protein stability of Aurora-A were correlated with increased PUM2 expression. This observation suggests that PUM2 can physically interact with Aurora-A, thereby preventing it from being targeted by APC/C^Cdh1^ for ubiquitination.

We speculated that PUM2 might bind to the amino-terminal A-box or the carboxy-terminal D-box of Aurora-A to prevent the recognition by APC/C^Cdh1^. To test this speculation, the PUM2-binding domain of Aurora-A was mapped by co-immunoprecipitation assay using anti-HA antibody. Flag-PUM2 was cotransfected with various HA-Aurora-A truncation mutants into cells for co-immunoprecipitation assay. Figure 4B shows that the Aurora-A(1–131) and Aurora-A(1–362) failed to associate with PUM2, and that the carboxy-terminal D-box of Aurora-A seemed to be as an essential determinant for the interaction between Aurora-A and PUM2. This suggests that PUM2 may protect Aurora-A from attack by APC/C^Cdh1^ on the D-box and thereby enhance its stability.

### Increase of Aurora-A kinase activity by PUM2 *in vitro*

**Figure 5:**
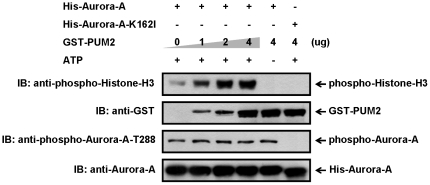
Increase of Aurora-A kinase activity by PUM2 in a dose-dependent manner. Recombinant His-tagged Aurora-A or kinase-inactive Aurora-A mutant protein (K162I) was incubated with the indicated amount of GST-PUM2 for 20 min at 30°C in kinase reaction buffer containing histone-H3 and ATP. The activity of Aurora-A was detected by immunoblotting using the anti-phospho-Aurora-A-T288 and the anti-phospho-Histone-H3 antibodies.

Our observations that PUM2 could stabilize Aurora-A suggest that PUM2 might serve as an upstream regulator of Aurora-A. In general, there are two routes by which protein kinases are regulated: one, through modulating its quantity in cells and two, through modulating the kinase activity. To determine the effect of PUM2 on activating Aurora-A, an *in vitro* kinase assay was performed to measure the activity of Aurora-A by using Histone-H3 as a substrate. Different amounts of purified GST-PUM2 recombinant proteins were incubated with His-tagged Aurora-A in kinase reaction buffer containing ATP. It was found that the phosphorylation of Histone-H3 was markedly increased by the addition of PUM2 in a dose-dependent manner (Figure 5). Although we could not exclude the possibility that PUM2 is also a downstream effector of Aurora-A during cell division, these results strongly argue that PUM2 is an upstream regulator of Aurora-A and that it not only serves as an Aurora-A stabilization factor but also serves as an Aurora-A-activating factor.

## Discussion

Here we report two main findings. First, the translational regulator, PUM2, is a cell cycle-regulated protein and a novel substrate of Aurora-A. Second, PUM2 can interact with Aurora-A to protect Aurora-A from APC/C^Cdh1^-mediated protein ubiquitination/degradation and to enhance the kinase activity of Aurora-A. This reveals the moonlight role of PUM2 in mitotic control, apart from its role in translational regulation during interphase.

Befitting that Aurora-A kinase plays a central role in cell division, the substrates of this kinase include various cell cycle regulatory proteins such as TPX2 [26], TACC3 [23], HURP [27], [28], Eg5 [29], CDC25B [30], Ajuba [5] and PAK1 [6]. Together, these substrates are presumed to be responsible for at least some of the cell cycle activities that have been ascribed to Aurora-A. It is imperative to identify any additional substrates and interacting proteins that may exist to complete the description of the role of Aurora-A in the regulation of mitosis. PUM2, a novel Aurora-A substrate that has been identified in this study, has a well-known role in the regulation of translation. Interestingly, our results suggest an additional novel role of PUM2 in serving as an upstream regulator of Aurora-A through physical interaction. The molecular mechanism of PUM2-mediated kinase regulation acts to both activate and stabilize Aurora-A. PUM2 could affect the cell cycle progression through regulation of Aurora-A.

**Figure 6:**
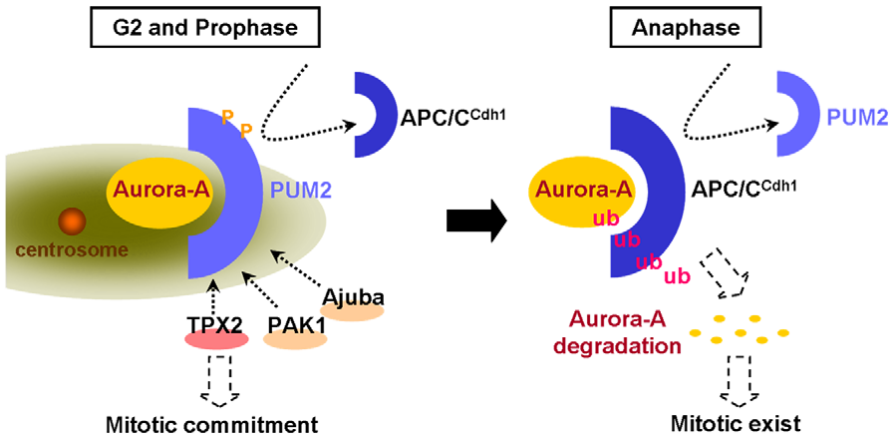
The mechanism by which PUM2 regulates the cell cycle progression. Through recruitment of the Aurora-A activator and by protecting Aurora-A from attack by APC/C^Cdh1^, PUM2 might trigger an increase in the amount of Aurora-A and increase its kinase activity dramatically, causing mitotic entry.

Overexpression of the PUM2 mutant, which failed to interact with Aurora-A, and depletion of PUM2 both led to the destabilization of Aurora-A (Figure 3). This was made evident by the low susceptibility of Aurora-A to ubiquitination in cells in which PUM2 was overexpressed (Figure 4A). Moreover, the carboxy-terminal D-box of Aurora-A was shown to be the region responsible for PUM2 binding (Figure 4B), suggesting that the binding of PUM2 might protect Aurora-A from being attack by APC/C^Cdh1^ (Figure 6). In a recent report, the mRNAs associated with PUM2 were systematically identified by the recovery of endogenously formed ribonucleoprotein complexes and the analysis of associated RNAs with DNA microarrays [31]. This study indicated that Aurora-A mRNA was not included in the transcripts that were reproducibly associated with PUM2. This precluded possible effects of PUM2 on Aurora-A mRNA translation. Moreover, it has been reported that APC/C^Cdh1^ is kept inactive in early mitosis and becomes active by the phosphorylation of cyclin-dependent kinase (cdk) only from late mitosis to G1 phase [32]. Why there is PUM2 to protect Aurora-A from being attack by APC/C^Cdh1^ in early mitosis? It is reported that timely destruction of the anaphase inhibitor, securin, by APC/C^Cdh1^ is regulated by the nucleocytoplasmic transport factors Rae1 and Nup98 [33]. Rae1 and Nup98 would form a complex with APC/C^Cdh1^ and securin during prometaphase. This suggested that the Rae1-Nup98 complex does not inhibit the destruction of securin by preventing the interaction between APC/C^Cdh1^ and securin, but by preventing the ubiquitination of APC/C^Cdh1^-bound securin. It has been proposed that when securin is restricted in the Rae1-Nup98 complex, it would prime cells for the sudden degradation of securin at the metaphase-anaphase transition [34]. In this study, we found that PUM2 could affect the ubiquitination of Aurora-A by forming a complex with Aurora-A. PUM2 might control the destruction of Aurora-A through a similar mechanism in regulating the degradation of securin, although it remains to be determined whether APC/C^Cdh1^ is in the PUM2-Aurora-A complex in early mitosis.

In addition to its role in enhancing protein stability, the physical interaction of PUM2 and Aurora-A is also required for the promotion of Aurora-A kinase activity (Figure 5). It has been shown that PUM2 binds to the C-terminal region of Aurora-A, the same region that has been identified as the interaction site for TPX2 with Aurora-A [4]. This suggests that PUM2 might mediate the kinase activity of Aurora-A through the same mechanism as TPX2. However, only substrate phosphorylation activity of Aurora-A was increased in the presence of PUM2, not its auto-phosphorylation activity (Figure 5). This suggests that the phenomenon observed might be due to another mechanism. PUM2 is a relatively large protein containing 1064 amino acids (theoretical molecular weight 114 kDa). Therefore, we speculated that the proper phosphorylation and accumulation of PUM2 at the centrosome might serve as a scaffold for the recruitment of other Aurora-A activators such as TPX2, PAK1 and Ajuba to regions that are proximal to the centrosomes, thereby triggering the rapid increase in the activity of Aurora-A (Figure 6). Moreover, it is interesting to note that PUM2 was localized in the centrosome from S-phase to metaphase. When the cells entered anaphase, PUM2 could not be detected in the centrosome, consistent with the time at which Aurora-A becomes unstable and with less kinase acitvity (Figure 1B). This indicated that PUM2 might be essential for mitotic entry by means of the regulation of Aurora-A. Through recruiting Aurora-A activators and protecting Aurora-A from attack by APC/C^Cdh1^, PUM2 might enhance the quantity and activity of Aurora-A dramatically, thereby causing mitotic entry. It will be interesting to learn if PUM2 competes or cooperates with the other, previously identified activators or destabilization factors of Aurora-A, and if Aurora-A interacts with more than one regulator at a time.

It has been shown that PUM2 is involved in the regulation of cell division as the other two factors, Maskin and CPEB, that are involved in regulating the translation of *Xenopus* maternal cyclin B1 mRNA. Besides, several studies also revealed that interphase components might be used to regulate mitosis, and many mitotic key regulators also have important functions during interphase. First, RanGTP, a protein that has a well-established function in nuclear trafficking in interphase, uses similar principles to regulate spindle assembly in mitosis as it does during interphase [35], [36]. Another interphase component, Lamin B, which is important for nuclear envelope assembly and in maintaining nuclear shape, has also been reported to play a role in mitosis. It can assemble into a matrix-like network during mitosis, and the formation of this mitotic matrix then tethers a number of spindle assembly factors, followed by the stimulation of microtubule assembly [37]. Moreover, the Aurora-A activators PAK1, HEF and Ajuba are present at focal adhesions and serve as downstream effectors of integrin during the process of cell migration. It is possible that disassembly of focal adhesion molecules during mitosis releases the protein complexes and results in the activation of Aurora-A [38].

In conclusion, all components in the PUM2-CPEB-Maskin protein complex are not only involved in translational regulation in interphase but also have roles in the progression of mitosis. This finding delineates an economical design that employs components that regulate both the progression of interphase and mitosis.

## Material and Methods

### Ethics statement

The rabbit work involved in generating antibody in this study was done according the animal use protocol of the Institutional Animal Care and Use Committee(IACUC)of Fu Jen Catholic University with ethics approval number: A9856.

### Construction of expression vectors, small interference RNA (siRNA) and Site-directed mutagenesis

Human PUM2 cDNA and Aurora-A were subcloned into pFLAG-CMV-2 vector (Sigma) and pcDNA3.0 (Invitrogen) to produce FLAG-tagged and HA-tagged plasmids. To generate GST fusion protein, PUM2 cDNA were subcloned to pGEX4T-2 vector (Amersham Pharmacia Biotech). Aurora-A cDNA were subcloned to pET29a vector (Novagen) to generate His-tagged protein. The sequences of the siRNAs were as follows: Aurora-A siRNA, 5′-GAGUCUACCUAAUUCUGGA-3′, and PUM2 siRNA, 5′-GCAUGGUAGAAUAUGUAUU-3′. Chemical synthesized 21 nt double strand siRNAs were purchased from Applied Biosystems. To generate siRNA-resistant form of PUM2 (siRNA-R-FLAG-PUM2), PCR-based mutagenesis (Quik-Change™ Site-directed mutagenesis kit, Stratagene) was used to achieve the desired mutations.

### Cell Culture, transfection, immunofluorescence analysis and *in situ* proximity ligation assay (PLA)

All cell lines were purchased from the American Type Culture Collection, and all cell culture-related reagents were purchased from Gibco-BRL. HEK293T human embryonic kidney cells and HeLa human cervical adenocarcinoma cells were grown in Dulbecco's modified Eagle's medium containing 10% FBS, 2 mM glutamine and100 unit/ml penicillin and streptomycin (Gibco-BRL). For maintaining CL_1–5_ lung cells, RPMI medium was used. Transfection of the cells was performed with Lipofectamine (Invitrogen) according to the manufacturer's instructions. For immunofluorescence analysis, cells cultured on cover-slips for 24 hrs were fixed with 4% formaldehyde for 30 min, permeabilized with 0.5% NP-40 for 5 min, and then stained with primary antibody at room temperature for 1 hr, followed by addition of fluorochrome-labeled secondary antibodies (Invitrogen) at room temperature for 50 min. For *In situ* proximity ligation assay (PLA), the Rabbit PLUS and Mouse MINUS Duolink *in situ* PLA kits (OLINK Bioscience) were used according to the manufacturer's protocol. PLA probes were diluted in 1% fetal calf serum and incubated in a pre-heated humidity chamber for 1 hr at 37°C, followed by hybridization, ligation, amplification and detection. The stained slides were observed using the Olympus FV1000 confocal fluorescence microscope.

### Preparation of cell lysates, dephosphorylation assay, immunoprecipitation and immunoblotting analysis

Cells were harvested, washed with phosphate-buffer saline (PBS), and lysed in extraction buffer [39]. After incubation at 4°C for 30 min, cellular debris was removed by centrifugation at 13,000 rpm for 30 min. Protein concentrations were determined using the Bradford assay (Bio-Rad). To perform the protein dephosphorylation experiment, lysates (50 µg) or the immunoprecipitation products were incubated with 400 U of λ phosphatase (New England Biolabs) in λ phosphatase reaction buffer with or without protein phosphatase inhibitor (Roche) at 30°C for 30 min. To increase the separation of the phosphorylated and unphosphorylated forms of the protein, equal amounts of treated and untreated total lysates were loaded side by side on a SDS-PAGE, and the acrylamide-to bisacrylamide ratio was adjusted from 29∶1 to 100∶1. For immunoprecipitation assay, 1 mg of total cell lysate was incubated with antibodies against target epitopes and Protein A/G-agarose beads (Santa Cruz) to immunoprecipitate the target proteins. The immunoprecipitation products were resolved by SDS-PAGE. The antibodies used in the present study were mouse monoclonal anti-FLAG M2 (Sigma), mouse monoclonal anti-HA (Sigma), mouse monoclonal anti-GAPDH (Santa Cruz), mouse monoclonal anti-β actin (Sigma), mouse monoclonal anti-Aurora A (BD), rabbit anti-phospho-Aurora-A-T288 (Cell Signaling Technology), mouse monoclonal anti-cyclin B1 (Upstate Biotechnology), rabbit anti-phospho-Histone-H3 (Upstate Biotechnology). For generating anti-PUM2 antibodies, recombinant GST-tagged PUM2 was purified as described in method of “Preparation of recombinant protein” and then injected into rabbit to raise polyclonal PUM2 antibodies. Quantification of the intensity of protein bands on immunoblotting analysis was performed with the Multi Gauge software (FUJI FILM).

### Preparation of recombinant protein, *In vitro* binding assay and *in Vitro* kinase reaction

pET29a-Aurora-A and pGEX4T2-PUM2 constructs were tramsformed in *E. Coli* BL21 (DE3). Recombinant His-tagged Aurora-A and GST-tagged PUM2 were induced for 4 hr at room temperature with 1 mM IPTG and purified from the soluble fraction by nickel-agarose (Qiagen) and Glutathione-Sepharose beads (Amersham Pharmacia Biotech). In *in vitro* binding assay, recombinant GST-tagged PUM2 (40 µg) on Glutathione-Sepharose beads was incubated with 100 µg purified recombinant His-tagged Aurora-A in binding buffer at 4°C for overnight. After incubation, the fusion protein-Sepharose complexes were washed with washing buffer. The bound proteins were eluted by boiling in the SDS sample buffer, and subjected to immunoblotting analysis. For the *in vitro* kinase reaction, the purified GST-tagged PUM2 (3 µg) or FLAG-tagged PUM2 immunoprecipitated from cell lysates was incubated with 3 µg of purified recombinant His-tagged Aurora-A in kinase buffer [40] containing [γ-^32^P]-ATP. After incubation at 30°C for 30 min, samples were subjected to SDS-PAGE and phosphorylated PUM2 were visualized by autoradiography.

### Cell-cycle synchronization and flow cytometry analysis

To synchronize cells at the G1/S boundary or in mitosis, exponentially cells were cultured in the presence of thymidine or nocodazole respectively, following the protocol as previously described [39]. Cells were released into cell cycle progression by removing the thymidine or nocodazole followed by incubation with fresh culture medium. At continuous time point, cells were collected and analyzed by immunoblotting and flow cytometry analysis. In the flow cytometry analysis, cells were fixed in 70% ethanol and store at 4°C overnight. Cells were then washed with PBS and treated with RNase A (0.1 mg/ml in PBS) for 5 min at room temperature. RNase A-treated cells were stained with propidium iodine (20 mg/ml) for at least 20 min at 4°C and analyzed by BD FACSCanto flow cytometry system.

### Cycloheximide inhibition assay

The turnover rates of proteins were determined using cycloheximide to inhibit protein synthesis. Cells treated with 50 µg/ml cycloheximide (Sigma) were harvested at different time point after removing the cycloheximide inhibition. Equal amounts of cell lysates were subjected to SDS-PAGE and analyzed by immunoblotting.

### Ubiquitination assay

HEK293T cells were transfected with FLAG-tagged Aurora-A alone or together with FLAG-tagged PUM2. Myc-tagged ubiquitin was also added to reveal Aurora-A ubiquitination. 24 hrs after transfection, cells were synchronized in the G2/M phase by nocodazole treatment for 16 hr. Subsequently, synchronized cells were released into cell cycle progression in the presence or absence of 25 µM proteasome inhibitor (MG132) for 9 hr [41]. Cells were harvested and analyzed by immunoblotting.

## Supporting Information

Figure S1 **Aurora-A can phosphorylate PUM2, leading to the electrophoretic mobility up-shift of PUM2.** HEK293T cells were transfected with FLAG-tagged PUM2 in combination with FLAG-tagged Aurora-A. The samples were immunoprecipitated using an anti-FLAG antibody to precipitate FLAG-tagged PUM2 from cell lysates followed by λ protein phosphatase treatment. A reaction with a protein phosphatase inhibitor was analyzed in parallel.
(TIF)

Figure S2 **Using** ***in vitro*** **kinase assay to confirm that PUM2 is the substrate of Aurora-A.** Different amounts of GST-tagged PUM2 was incubated, either alone or in combination with recombinant His-tagged Aurora-A, in the presence of [γ-32P]-ATP. The samples were electrophoresed using SDS-PAGE and transferred to a PVDF membrane. They were then either autoradiographed or immunoblotted. Phospho-PUM2 radioactivity could be detected in the *in vitro* kinase reaction contain 0.5 µg of substrate (PUM2) and only in the presence of Aurora-A.
(TIF)

Figure S3 **PUM2Δ434–740, lacking the S motif and the sequence preceding the PUM-HD motif, were not able to bind to Aurora-A.** HEK293T cells were transfected with HA-tagged Aurora-A in combination with wild-type FLAG-tagged PUM2 or FLAG-tagged PUM2 deleted mutant, PUM2Δ434–740. The cell lysates were immunoprecipitated with an anti-HA antibody and then immunoblotted with an anti-FLAG antibody to detect PUM2.
(TIF)

Figure S4 **The stability of Aurora-A is regulated by Cdh1 but not Cdc20.** HEK293T cells were transfected with FLAG-tagged Aurora-A, either alone or with different amounts of FLAG-taggedCdh1 (A) or Cdc20 (B). An empty vector was added to bring the total amount to 4 ug of DNA. The cell lysates were subsequently immunoblotted.
(TIF)
