# Supplementary figures and images for: A Translational Regulator, PUM2, Promotes Both Protein Stability and Kinase Activity of Aurora-A
Source: PLoS One. 2011 May 11;6(5):e19718. doi: 10.1371/journal.pone.0019718 (PMC3092770; doi:10.1371/journal.pone.0019718)

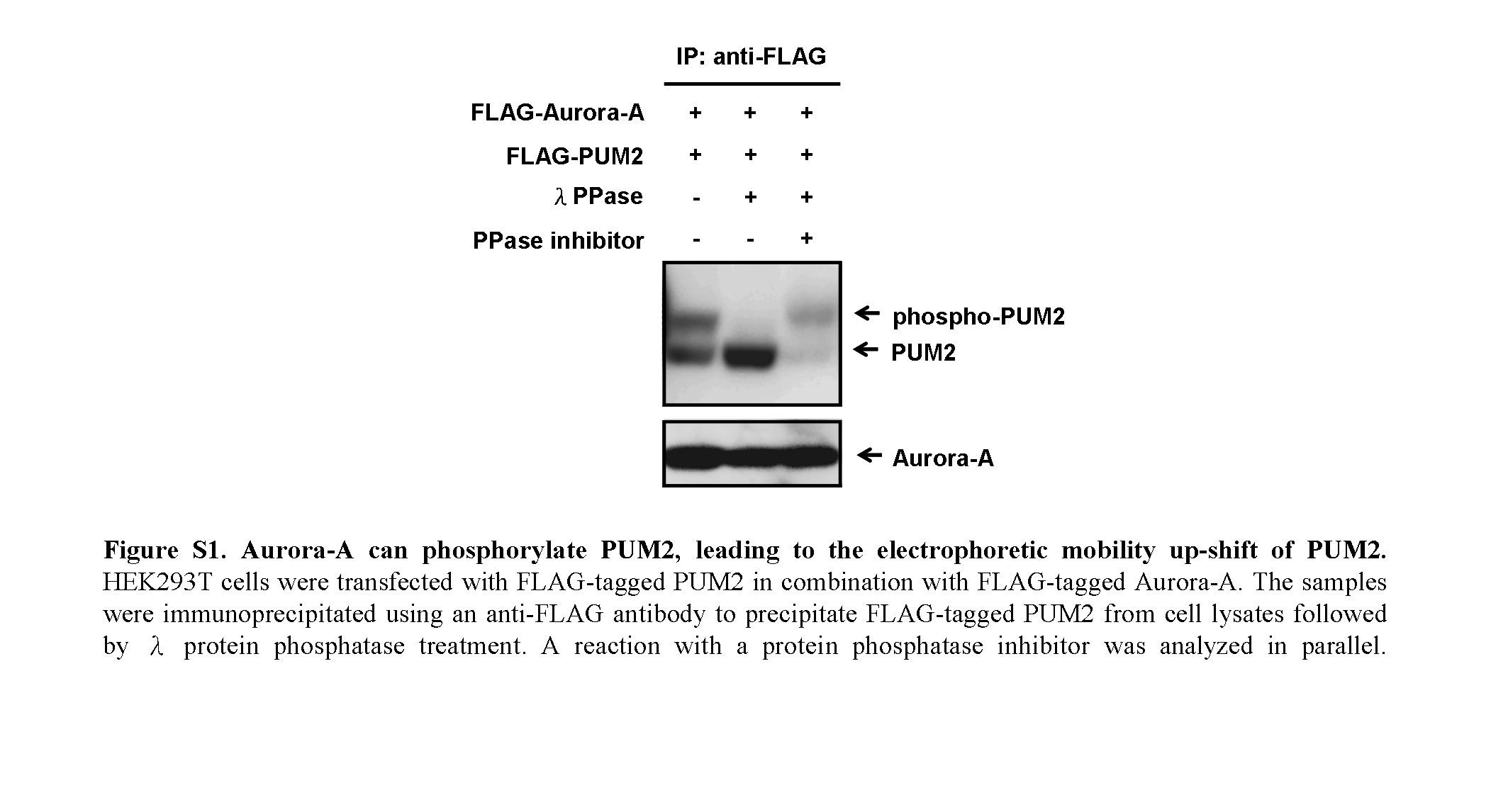

Supplement: Figure S1 — Aurora-A can phosphorylate PUM2, leading to the electrophoretic mobility up-shift of PUM2. HEK293T cells were transfected with FLAG-tagged PUM2 in combination with FLAG-tagged Aurora-A. The samples were immunoprecipitated using an anti-FLAG antibody to precipitate FLAG-tagged PUM2 from cell lysates followed by λ protein phosphatase treatment. A reaction with a protein phosphatase inhibitor was analyzed in parallel. (TIF) [file pone.0019718.s001.tif]

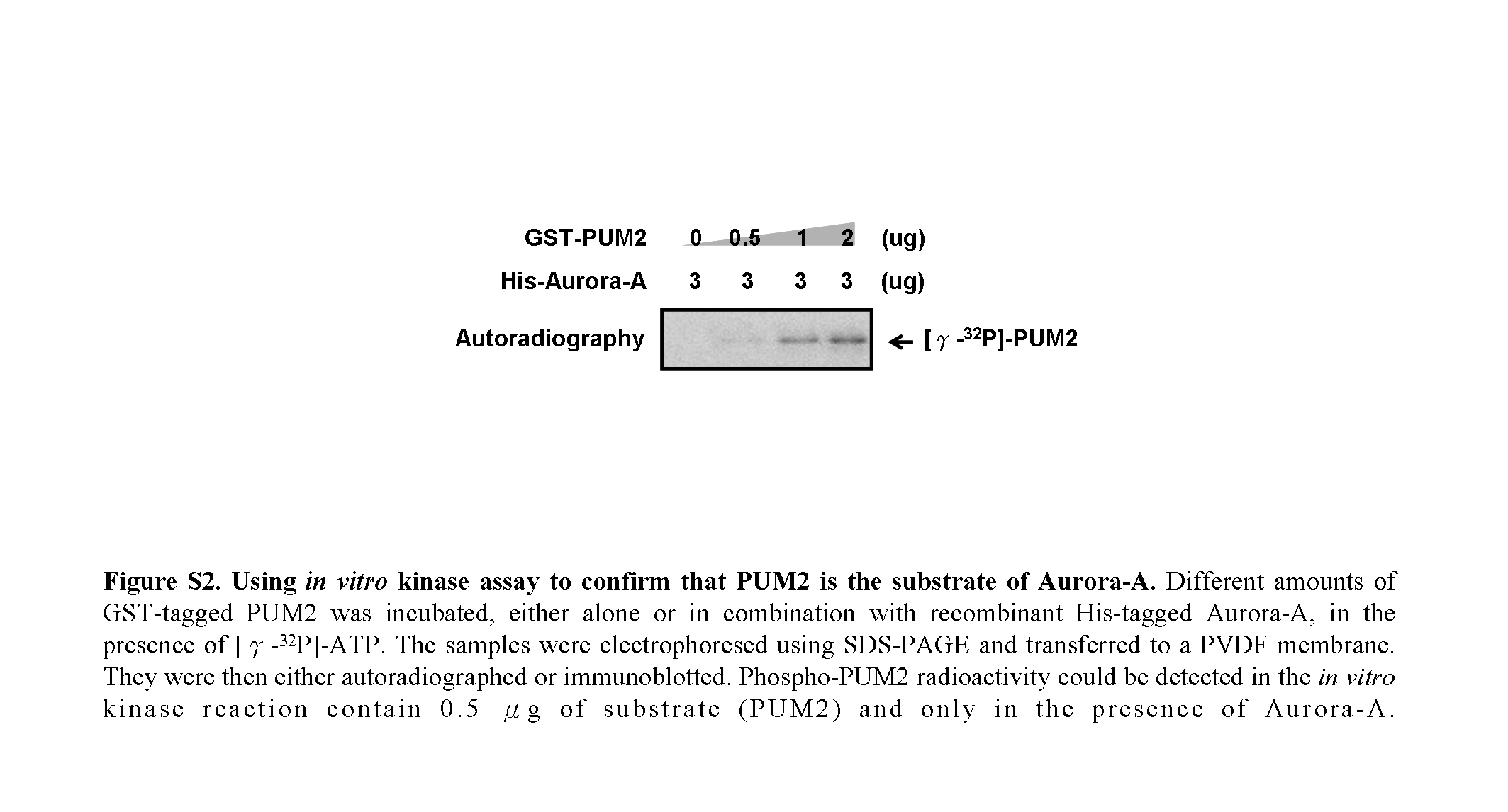

Supplement: Figure S2 — Using in vitro kinase assay to confirm that PUM2 is the substrate of Aurora-A. Different amounts of GST-tagged PUM2 was incubated, either alone or in combination with recombinant His-tagged Aurora-A, in the presence of [γ-32P]-ATP. The samples were electrophoresed using SDS-PAGE and transferred to a PVDF membrane. They were then either autoradiographed or immunoblotted. Phospho-PUM2 radioactivity could be detected in the in vitro kinase reaction contain 0.5 µg of substrate (PUM2) and only in the presence of Aurora-A. (TIF) [file pone.0019718.s002.tif]

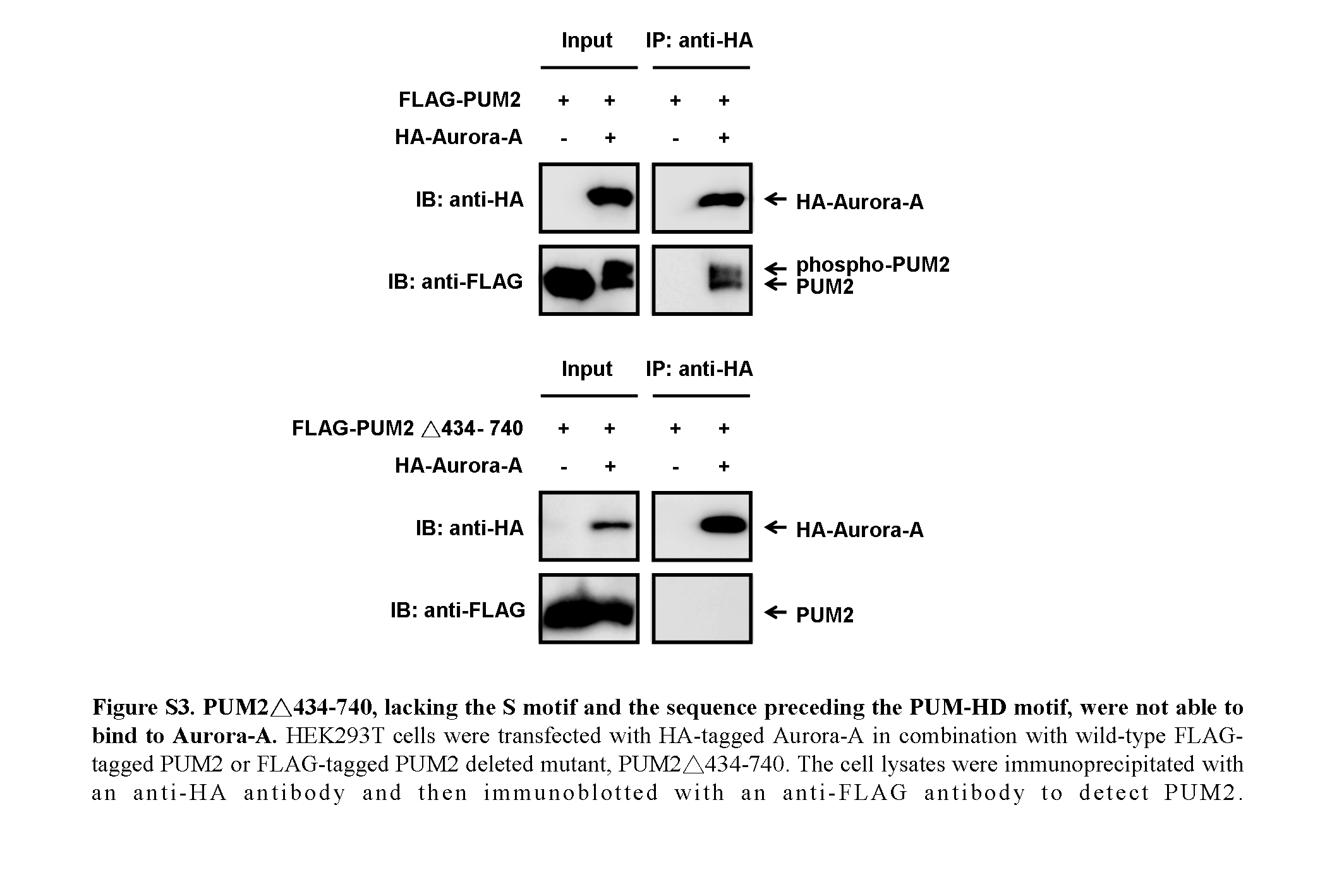

Supplement: Figure S3 — PUM2Δ434–740, lacking the S motif and the sequence preceding the PUM-HD motif, were not able to bind to Aurora-A. HEK293T cells were transfected with HA-tagged Aurora-A in combination with wild-type FLAG-tagged PUM2 or FLAG-tagged PUM2 deleted mutant, PUM2Δ434–740. The cell lysates were immunoprecipitated with an anti-HA antibody and then immunoblotted with an anti-FLAG antibody to detect PUM2. (TIF) [file pone.0019718.s003.tif]

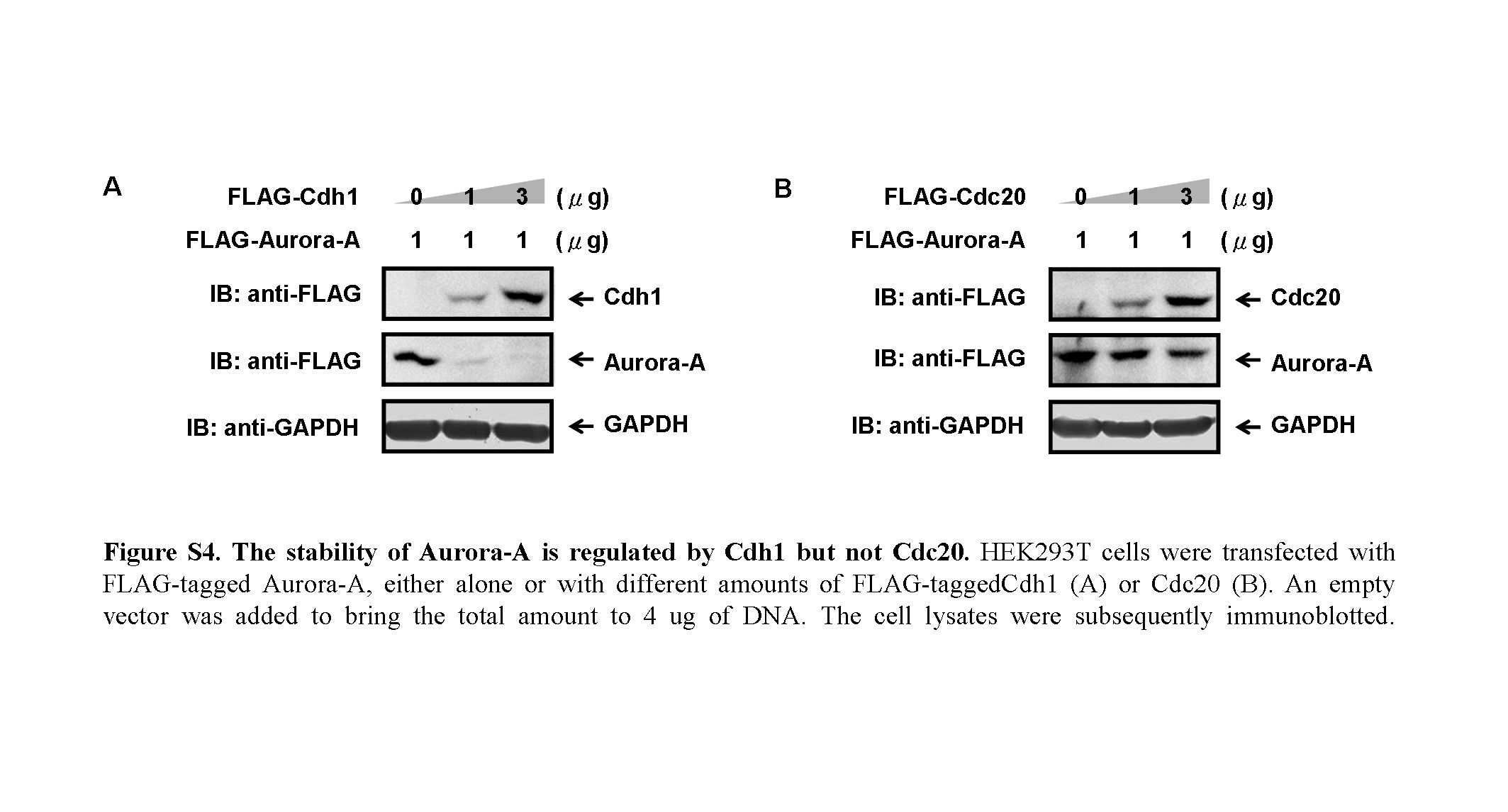

Supplement: Figure S4 — The stability of Aurora-A is regulated by Cdh1 but not Cdc20. HEK293T cells were transfected with FLAG-tagged Aurora-A, either alone or with different amounts of FLAG-taggedCdh1 (A) or Cdc20 (B). An empty vector was added to bring the total amount to 4 ug of DNA. The cell lysates were subsequently immunoblotted. (TIF) [file pone.0019718.s004.tif]
